# Effect of low-dose gaseous ozone on pathogenic bacteria

**DOI:** 10.1186/1471-2334-12-358

**Published:** 2012-12-18

**Authors:** Belchor Fontes, Ana Maria Cattani Heimbecker, Glacus de Souza Brito, Silvia F Costa, Inneke M van der Heijden, Anna S Levin, Samir Rasslan

**Affiliations:** 1 3rd Division of Clinical Surgery and Laboratory of Medical Investigation (LIM-62), Hospital das Clínicas, University of São Paulo, São Paulo, Brazil; 2 Laboratory of Medical Investigation (LIM-62), Hospital das Clínicas, University of São Paulo, São Paulo, Brazil; 3 Clinical Immunology and Allergy Division, Hospital das Clínicas, University of São Paulo, São Paulo, Brazil; 4 Department of Infectious Diseases and LIM54, University of São Paulo, São Paulo, Brazil; 5 Laboratory of Medical Investigation (LIM-54), Hospital das Clínicas, University of São Paulo, São Paulo, Brazil; 6 Departments of Infectious Diseases and Nosocomial Infection Control and LIM54, University of São Paulo, Rua Banibas, 618, São Paulo, SP, 05460-010, Brazil; 7 Department of Surgery, University of São Paulo, São Paulo, Brazil

**Keywords:** Ozone, Resistant bacteria, *in vitro* study

## Abstract

**Background:**

Treatment of chronically infected wounds is a challenge, and bacterial environmental contamination is a growing issue in infection control. Ozone may have a role in these situations. The objective of this study was to determine whether a low dose of gaseous ozone/oxygen mixture eliminates pathogenic bacteria cultivated in Petri dishes.

**Methods:**

A pilot study with 6 bacterial strains was made using different concentrations of ozone in an ozone-oxygen mixture to determine a minimally effective dose that completely eliminated bacterial growth. The small and apparently bactericidal gaseous dose of 20 μg/mL ozone/oxygen (1:99) mixture, applied for 5min under atmospheric pressure was selected. In the 2^nd^ phase, eight bacterial strains with well characterized resistance patterns were evaluated *in vitro* using agar-blood in adapted Petri dishes (10^5^ bacteria/dish). The cultures were divided into 3 groups: 1- ozone-oxygen gaseous mixture containing 20 μg of O_3_/mL for 5 min; 2- 100% oxygen for 5 min; 3- baseline: no gas was used.

**Results:**

The selected ozone dose was applied to the following eight strains: *Escherichia coli*, oxacillin-resistant *Staphylococcus aureus*, oxacillin-susceptible *Staphylococcus aureus*, vancomycin-resistant *Enterococcus faecalis*, extended-spectrum beta-lactamase-producing *Klebsiella pneumoniae*, carbapenem-resistant *Acinetobacter baumannii*, *Acinetobacter baumannii* susceptible only to carbapenems, and *Pseudomonas aeruginosa* susceptible to imipenem and meropenem. All isolates were completely inhibited by the ozone-oxygen mixture while growth occurred in the other 2 groups.

**Conclusion:**

A single topical application by nebulization of a low ozone dose completely inhibited the growth of all potentially pathogenic bacterial strains with known resistance to antimicrobial agents.

## Background

Numerous alternatives for the treatment of chronically infected wounds have been described in the literature. The real role of various agents, topically applied, remains undetermined [1-3].Topical application of antibiotics has shown limited efficacy in the management of infected wounds, without conclusive results [4,5]. In the last several years, various *in vitro* and *in vivo* experimental and clinical studies have investigated the bactericidal effect of the topical application of ozone in different situations, including the management of infected wounds [6-8]. O_3_ is a potent oxidant and an important disinfectant, acting on microorganisms by means of oxidation of their biological material [9]. It has been reported that O_3_ can be employed as a bactericidal agent under various forms, such as ozonized saline solution [10], ozonized water [11], ozonized oil [7], ozone associated with other substances [12], and more frequently the gaseous O_3_/O_2_ mixture [13]. The topical use of O_3_ has also been reported for environment decontamination in diverse situations: in agriculture for food decontamination [14], in odontology [15], and in clinical settings such as in the treatment of infected wounds [7]. Gaseous ozone has also been potentially considered for the disinfection of the hospital environment, which can be a source of microorganisms for patients [16]. However, a minimal effective antibacterial dose of gaseous ozone for topical application has not been clearly determined [17].

The objective of this study was to determine whether a low dose of O_3_, in a gaseous O_3_/O_2_ mixture, applied to Petri dishes containing bacterial culture completely eliminates the growth of different bacterial strains. The strains chosen in this study were those that were important pathogens of nosocomial and community-acquired infections and those with known mechanisms of antimicrobial resistance.

## Methods

This study was performed at the 3^rd^ Division of Clinical Surgery and Laboratory of Medical Investigation (LIM-54 and 62), of the Hospital das Clínicas of the University of São Paulo School of Medicine. The project was approved by the Ethics Committee of the Institution.

**Figure 1:**
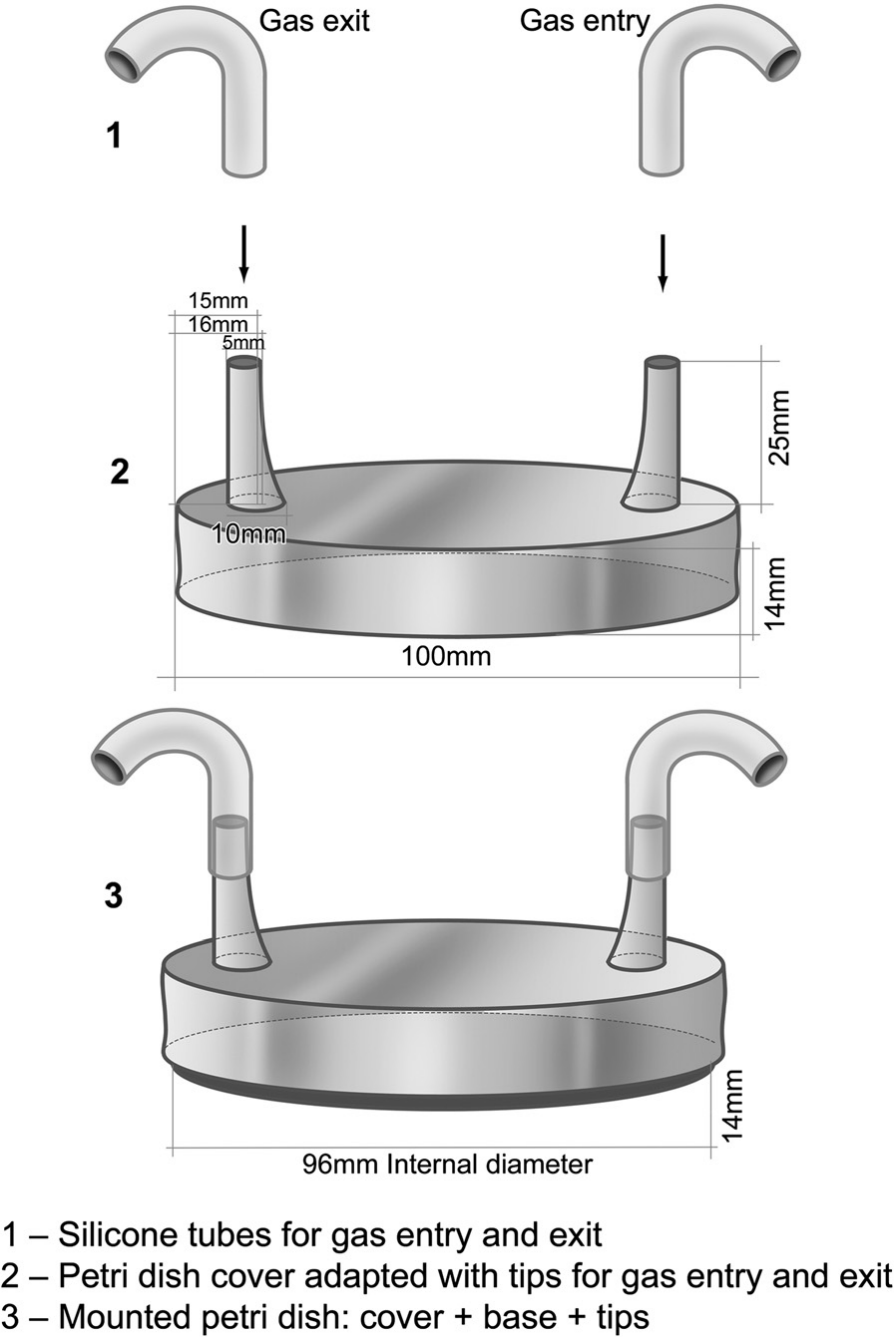
Schematic representation of adapted Petri dish with the addition of two special tips in order to allow continuous gas entry and exit.

**Figure 2:**
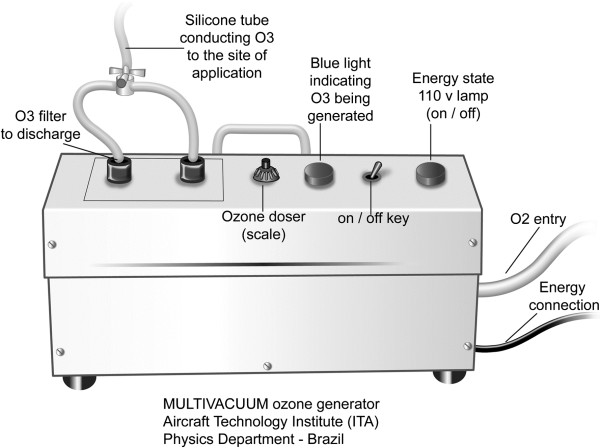
**Schematic representation of a medicinal O**_**3**_**generator equipped with an oxygen flow controller with 0.01 min precision, in which a controlled oxygen flow passes through a glass cylinder and is exposed to an electric discharge by a dielectric barrier with controlled potency and voltage.**

For bacterial culture, Petri dishes (90 mm internal diameter) were adapted with the addition of two special tips in order to allow continuous gas entry and exit (Figure 1). The study was done in two phases: a pilot test (1^st^ phase) to determine the dose of O_3_/O_2_ to be later employed in the evaluation of its bactericidal activity when applied to different bacterial strains (2^nd^ phase). In both phases, bacterial strains were inoculated on agar-blood in the adapted Petri dishes, at a concentration of 10^5^ bacteria/dish. Ozone gas was produced from medicinal oxygen (liquid oxygen with a degree of purity of 98%) by means of a medicinal O_3_ generator provided by the Gas Department of the Aircraft Technology Institute (Instituto de Tecnologia da Aeronáutica – ITA, Brazilian Army, São José dos Campos, Brazil). This instrument was equipped with an oxygen flow controller (MKS – Type 1179ª) with 0.01 min precision, in which a controlled oxygen flow passes through a glass cylinder and is exposed to an electric discharge by a dielectric barrier with controlled potency and voltage (Figure 2). The electric discharge generates O_3_ and its concentration can be measured. The final product is a gaseous O_3_/O_2_ mixture, in which the concentration is regulated by variation of oxygen flow and the voltage applied to the electrodes.

### First phase (pilot test)

In this phase, *Escherichia coli* – ATCC:35218, *Staphylococcus aureus* susceptible to oxacillin – ATCC:25923, and *Pseudomonas aeruginosa* susceptible to imipenem and meropenem – ATCC:27853 were inoculated on agar-blood in Petri dishes and incubated aerobically at 35±2°C for 18 to 24 hours. A standardized inoculum was prepared using the direct colony suspension by making a saline suspension of isolated colonies selected from blood agar plates. Each bacterial suspension was adjusted to 0.5 McFarland turbidity standard (1 to 2 x 10^8^ CFU/mL) using a photometric device (colorimeter Vitek®1, BioMérieux, Etoile, France). The 0.5 McFarland suspension was diluted 1:1000 in sterile saline, resulting in a tube containing approximately 10^5^ CFU/mL. An aliquot of 10μL of this suspension was inoculated in another blood agar plate (final concentration of 10^3^ CFU/mL). Then the dishes were then subjected to decreasing doses of O_3_, starting with 100 μg/mL. Initially, the plates were exposed for 60 min. The time and the dose were subsequently decreased. The minimal dose and time required to completely prevent bacterial growth of the three microorganisms was determined (20 μg/mL for 5 min). O_3_ was then applied at the dose of 15 μg/mL for 5 min to six bacterial isolates (*Escherichia coli* – ATCC: 25922; *Staphylococcus aureus* resistant to oxacillin –ATCC:29213; *Staphylococcus aureus* susceptible to oxacillin – ATCC:25923; *Enterococcus faecalis* resistant to vancomycin – ATCC:51299; *Klebsiella pneumoniae* – ESBL-producer, susceptible only to carbapenems – clinical isolate from a patient, obtained as part of standard clinical care; and *Pseudomonas aeruginosa* – susceptible to imipenem and meropenem – ATCC:27853). The experiment was repeated four times for each microorganism, and the effects were evaluated.

### Second phase

For this phase of the study, eight potentially pathogenic bacteria strains were included, comprising the six employed in the first phase plus *Acinetobacter baumannii* susceptible only to carbapenem – ATCC:19606 and *Acinetobacter baumannii* resistant to carbapenem – clinical isolate from a patient, obtained as part of standard clinical care. Each strain was inoculated on agar-blood in four Petri dishes, and the colony forming units (CFUs) were counted at 24 h and 48 h following the treatment applied to each group. Three study groups were employed: the experimental group treated with 1% O_3_/99% O_2_ gas, corresponding to 20 μg of O_3_/mL (O_3_-group); and two control groups, including one treated with 100% oxygen (O_2_-group), and one called the Baseline group in which no gas was used.

Each bacterial strain was inoculated into four adapted Petri dishes, and 10 min later each dish (except for the Baseline group) received gas nebulization for 5 min, under atmospheric pressure. Thereafter, each dish was incubated at 37°C for 48 h. Colony counts were performed 24 h and 48 h following inoculation.

Statistical analysis of the results was performed using the Mann–Whitney Test to compare the global results obtained for the O_2_-group with those of the Baseline group after 24 and 48 h. The Kruskal-Wallis test was employed to compare the CFU counts of each bacterial strain with those of other strains, either within the same group, or of other groups. A p value < 0.05% was considered significant.

## Results

### Pilot study

The initial tests with the *E*. *coli*, *S*. *aureus*, and *P*. *aeruginosa* strains demonstrated that doses greater than or equal to 20 μg of O_3_/mL for 5 min totally prevented the growth of these three bacterial strains. However when 15 μg of O_3_/mL was applied for 5 min and the effect was evaluated on six bacterial strains, a low number of CFUs were present 48 h after inoculation for two of the six tested strains (Table 1). Therefore, 20 μg of O_3_/mL in the O_3_/O_2_ gaseous mixture for 5 min was chosen as the dose for the 2^nd^ phase.

**Table 1:** **Bacterial isolates submitted to an O**_**3**_/**O**_**2**_**gaseous mixture to determine the effect on the *in vitro *growth of bacteria**

**Bacterial strains exposed to 15μg/mL of O_3_ (in a O_3_/O_2_ gaseous mixture – 1%/ 99%) for 5 min**	**Growth at 48 hours (CFU)**			
	**P1**	**P2**	**P3**	**P4**
1= *Escherichia coli* – ATCC: 25922	**0**	**0**	**0**	**0**
2= *Staphylococcus aureus* resistant to oxacillin –ATCC:29213	**5**	**0**	**0**	**0**
3= *Staphylococcus aureus* susceptible to oxacillin – ATCC:25923	**0**	**0**	**0**	**0**
4= *Enterococcus faecalis* resistant to vancomycin – ATCC:51299	**0**	**0**	**0**	**0**
5= *Klebsiella pneumoniae* – ESBL-producer, susceptible only to carbapenems – Clinical isolate from a patient	**0**	**0**	**0**	**0**
6= *Pseudomonas aeruginosa* – susceptible to imipenem and meropenem – ATCC:27853	**9**	**1**	**0**	**0**

(Pilot study).

CFU: Colony-forming units: each experiment was repeated 4 times (P1 to P4).

### Second phase

The results are expressed as bacterial strain CFUs per experiment. All experiments for the O_3_-group showed complete inhibition of bacterial growth for all strains at 24 h and 48 h. This was statistically significant when comparing O_3_-group with each other group (p<0.014 for each bacterial strain). No difference in bacterial counts (p: 0.80) was noticed between the other two groups (Table 2). In Baseline and O_2_-groups, *Acinetobacter baumannii* resistant to carbapenems, presented CFU counts at 24 h and 48 h significantly greater (*p*<0.05) than all those of the other strains, except *Enterococcus faecalis*. For each bacterial strain, no significant difference was observed in CFU counts at 24 h compared to that at 48 h.

**Table 2:** **Bacterial *in vitro *growth, at 24 hours and 48 hours, of isolates submitted to an O**_**3**_/**O**_**2**_**gaseous mixture** (**O**_**3**_**group**), **to 100**% **O**_**2**_ (**O**_**2**_**group**) **and not submitted to gas treatment** (**Baseline group**)

**Bacterial strains**	**Culture duration**	**CFU / dish**											
		**O**_**3**_**Group**				**O**_**2**_**Group**				**Baseline Group**			
		**Plates (P)**				**Plates (P)**				**Plates (P)**			
		**P1**	**P2**	**P3**	**P4**	**P1**	**P2**	**P3**	**P4**	**P1**	**P2**	**P3**	**P4**
1= *Escherichia coli* – ATCC:25922	24 h	0	0	0	0	83	68	59	73	58	66	65	76
	48 h	0	0	0	0	78	69	58	61	57	68	62	80
2= *Staphylococcus aureus* resistant to oxacillin –ATCC:29213	24 h	0	0	0	0	94	81	80	55	98	83	104	95
	48 h	0	0	0	0	88	74	85	49	75	89	104	90
3= *Staphylococcus aureus* susceptible to oxacillin – ATCC:25923	24 h	0	0	0	0	72	45	82	68	65	44	91	76
	48 h	0	0	0	0	70	47	75	69	66	39	94	73
4= *Enterococcus faecalis* resistant to vancomycin – ATCC: 51299	24 h	0	0	0	0	69	64	201	75	73	100	105	71
	48 h	0	0	0	0	79	78	207	82	68	97	106	57
5= ESBL producing *Klebsiella pneumoniae* susceptible only to carbapenems –clinical isolate from a patient.	24 h	0	0	0	0	65	75	153	71	87	113	117	80
	48 h	0	0	0	0	68	81	135	69	96	88	108	80
6= *Acinetobacter baumannii* resistant to carbapenem – clinical isolate from a patient.	24 h	0	0	0	0	226	205	201	162	158	165	159	206
	48 h	0	0	0	0	214	196	171	137	135	162	130	185
7= *Acinetobacter baumannii* susceptible only to carbapenem – ATCC:19606	24 h	0	0	0	0	70	60	58	70	63	65	63	67
	48 h	0	0	0	0	69	61	52	63	65	69	62	64
8= *Pseudomonas aeruginosa* susceptible to imipenem and meropenem-ATCC:27853	24 h	0	0	0	0	155	68	138	94	82	85	88	65
	48 h	0	0	0	0	110	79	97	94	83	72	66	69

ESBL: Extended-spectrum beta-lactamase; CFU: Colony-forming units; each experiment was repeated 4 times (Plates: P1 to P4).

## Discussion

The eight bacterial strains employed in this study were selected to represent pathogenic bacteria commonly present in patients with severe nosocomial infections, with known resistance to antibiotics. Our results showed that the application of a low dose of gaseous ozone completely prevented the *in vitro* growth of all bacterial strains. On the other hand, in both control groups, bacterial growth occurred in all eight bacterial strains and treatment with 100% O_2_ had no effect on bacterial proliferation, compared with the Baseline group.

In the 1960s, Scott *et al*., using topical application of ozonized saline, showed that approximately 2 × 10^7^ molecules of O_3_ per bacterium provoked 50% death [10]. They attributed this to O_3_ reacting with lipid double bonds, thus leading to bacterial wall lysis and bacterial cell content extravasation [10]. By entering the cell, O_3_ promotes oxidation of nucleic and amino acids; and cell lysis depends on the extent of these reactions [1].

The culture medium employed in this study was agar-blood in Petri dishes. The use of agar for bacterial culture in Petri dishes is usual practice in microbiology, including the evaluation of bactericidal effects of different substances, such as ozone. An *in vitro* study aiming at decontamination with prolonged (4 h) application of gaseous O_3_ (2 ppm), revealed a reduction of viability of various bacteria, such as *E*. *coli*, *S*. *aureus*, *Serratia liquefaciens*, and *Listeria innocula*, suggesting a disinfectant effect of O_3_. The bacteria were cultured on agar in Petri dishes as well as in other culture media, and the author considered agar as the best culture media for measuring the efficacy of O_3_[14].

Pereira *et al*. reported that application of a gaseous O_3_/O_2_ mixture (0.4%/99.6%) for 1 h, at constant pressure and flow (11 mm Hg and 2 L/min, respectively) and controlled temperature, in plates containing 10^4^ CFU/mL of *E*. *coli*, *S*. *aureus*, and *P*. *aeruginosa* led to total inhibition of growth of these bacteria [18]. Compared to this study, the O_3_ concentration in the gaseous O_3_/O_2_ mixture in the present study was 2.5 times greater, the duration of application was much shorter (1/12), and the gas flow was half (1 L/min). Furthermore, in the present study, potentially pathogenic bacteria with higher inoculums and known antimicrobial resistance were selected. Due to these differences, comparison of these studies is not viable. Other *in vitro* studies involving gaseous ozone have been performed but cannot be compared with our study as they involve *Thichophyton* spp. [19], mutans streptococci [20] and *Listeria innocua*[21].

The potential of our findings is interesting. The hospital environment has been increasing implicated in the transmission of resistant bacteria such as methicillin-resistant *S*. *aureus* and enterococci [22,23]. Environmental cleaning and the application of hydrogen peroxide in the environment have recently deserved attention [24,25] and ozone may have a similar use. In 1973, Broadwater *et al*. determined the minimum dose of O_3_ dissolved in water (ozonized water) needed to eliminate the growth of three bacterial species when applied for 5 min. They observed that 0.12 mg/L of ozone was lethal for *Bacillus cereus*; and that 0.19 mg/L was lethal for *Bacillus megaterium* and *E*. *coli*[11]. Although O_3_ dissolved in water was employed for surface decontamination, there was no clear definition of a minimum effective dose for its application. Likewise, there is no clear dose for O_3_ in the form of an aerosol (O_3_ dissolved in air) to be employed for surface decontamination of scientific instruments [26,27]. Also focusing on environment decontamination, Li analyzed the resistance of various bacteria exposed to O_3_ for surface disinfection, and pointed out the importance of the species (*E*. *coli* was more susceptible) and of the O_3_ dose (concentration × time of exposure) on resistance to O_3_^29^. In the present study the dose of O_3_ employed totally prevented the growth of all bacterial strains although *Acinetobacter baumannii* had a greater inoculum than the other bacterial strains. A concern involving the use of environmental O_3_ for environmental disinfection is its toxicity, especially to the lungs [28] as the epithelial lining fluid has a relatively poor antioxidant capacity when compared with the blood. To enable the use of ozone in the hospital environment, exposure of patients and healthcare workers to inhalation would have to be avoided.

Another potential use for ozone is in the treatment of infected wounds. A clinical study reported that in the case of superficial wounds with antibiotic resistant sepsis following trauma and surgery, the application of O_3_/O_2_ resulted in wound healing and control of sepsis [8]. Sanchez *et al*. reported the efficient management of diabetic foot with gaseous O_3_/O_2_ application [29]. In a clinical prospective study [30], 61 patients with “diabetic foot” infections were randomized into two groups: topical gaseous O_3_ application (80μ/mL maintained during 20min/session) + conventional (debridement + wound dressing) *vs* placebo (O_2_ treatment). Although in the whole population the wound closure in the ozone group *vs* placebo (41% vs 33%) was not significant, it was observed that in the 34 patients who completed the study (16 of O_3_ and 18 placebo) the wound closure was significantly higher in the O_3_ group (81% vs 44%); and for patients with wounds ≤ 5cm^2^ the total closure was higher in the O_3_ group when compared with placebo (100% vs 50%; p=0.006). This suggested that O_3_ was superior to conventional treatment. However it is difficult to draw conclusions from such a small study. A point of concern is the toxicity of ozone to the skin. The skin is protected against oxidative stress by a variety of antioxidants [31], but chronic exposure to O_3_ can be deleterious to the skin, especially to the stratum corneum, leading to a cascade of effects in the deeper layers. Brief topical exposures of O_3_, however, have been shown to be non-toxic [31].

Our study presents the following limitations: it is a preliminary evaluation and focuses on the *in vitro* effect of a minimal dose of ozone applied in Petri dishes containing bacteria seeded superficially on the Agar medium. It is not yet clear how well our findings may translate into clinical practice in which factors such as variable blood flow, with ischemia, necrotic tissue and high bacterial burdens may play an important role, especially in the diabetic foot.

## Conclusions

In conclusion, the results of the present *in vitro* study showed that a dose of 20 μg of O_3_/mL in a gaseous O_3_/O_2_ mixture (1% O_3_/99% O_2)_, in a single topical application by nebulization for 5 min under atmospheric pressure, effectively inhibited the growth of all potentially pathogenic bacterial strains with known antimicrobial resistance.

## Competing interests

None of the authors have competing interests concerning this study.

## Authors’ contributions

Belchor Fontes- study design, analysis of data, and writing of manuscript. Ana Maria Cattani Heimbecker- laboratory work. Glacus de Souza Brito- conception of study. Silvia F. Costa- laboratory supervision. Inneke M. van der Heijden- laboratory work. Anna S. Levin- analysis of data, critical review of manuscript. Samir Rasslan: final approval of the manuscript. All authors read and approved the final manuscript.

## Funding

This study did not receive external funding.

## Pre-publication history

The pre-publication history for this paper can be accessed here:

http://www.biomedcentral.com/1471-2334/12/358/prepub
